# Spin Crossover in Nickel(II) Tetraphenylporphyrinate via Forced Axial Coordination at the Air/Water Interface

**DOI:** 10.3390/molecules26144155

**Published:** 2021-07-08

**Authors:** Alexander V. Shokurov, Daria S. Kutsybala, Andrey P. Kroitor, Alexander A. Dmitrienko, Alexander G. Martynov, Yulia Yu. Enakieva, Aslan Yu. Tsivadze, Sofiya L. Selektor, Yulia G. Gorbunova

**Affiliations:** 1 Frumkin Institute of Physical Chemistry and Electrochemistry, Russian Academy of Sciences, Leninsky pr. 31-4, 119071 Moscow, Russia; dariakutsybala@gmail.com (D.S.K.); andreykroytor96@gmail.com (A.P.K.); martynov.alexandre@gmail.com (A.G.M.); yulia.enakieva@gmail.com (Y.Y.E.); Atsiv43@mail.ru (A.Y.T.); sofs@list.ru (S.L.S.); 2 Department of Chemistry, Lomonosov Moscow State University, Leninskie Gory, 1-3, 119991 Moscow, Russia; das200261@gmail.com; 3 Kurnakov Institute of General and Inorganic Chemistry, Russian Academy of Sciences, Leninsky pr. 31, 119991 Moscow, Russia

**Keywords:** nickel porphyrinate, spin crossover, ruthenium phthalocyaninate, supramolecular, axial coordination, air/water interface, Langmuir monolayer, optical response

## Abstract

Coordination-induced spin crossover (CISCO) in nickel(II) porphyrinates is an intriguing phenomenon that is interesting from both fundamental and practical standpoints. However, in most cases, realization of this effect requires extensive synthetic protocols or extreme concentrations of extra-ligands. Herein we show that CISCO effect can be prompted for the commonly available nickel(II) tetraphenylporphyrinate, **NiTPP**, upon deposition of this complex at the air/water interface together with a ruthenium(II) phthalocyaninate, **CRPcRu(pyz)_2_**, bearing two axial pyrazine ligands. The latter was used as a molecular guiderail to align Ni···Ru···Ni metal centers for pyrazine coordination upon lateral compression of the system, which helps bring the two macrocycles closer together and forces the formation of Ni–pyz bonds. The fact of Ni(II) porphyrinate switching from low- to high-spin state upon acquiring additional ligands can be conveniently observed in situ via reflection-absorption UV-vis spectroscopy. The reversible nature of this interaction allows for dissociation of Ni–pyz bonds, and thus, change of nickel cation spin state, upon expansion of the monolayer.

## 1. Introduction

Axial coordination of molecules and anions to metal centers in tetrapyrrole compounds represents one of the generally acknowledged approaches to control photophysical and redox properties of these compounds, occurring in a large number of natural processes and technical applications [1]. It ranges from bonding of oxygen to the heme complex in hemoglobin of living beings [2,3] to formation of man-made molecular systems employing nanocarbons [4,5] and organic dyes [6,7] as extra-ligands. This coordinating ability of cation in a tetrapyrrolic environment can be exploited for development of coordination polymers of different dimensionalities and sophisticated bioinspired architectures [8,9,10,11]. Axial coordination can improve photosensitizing efficiency of tetrapyrrole-based anti-cancer drugs [12], modulate redox-activity of cytochromes and alter extracellular electron transfer [13], affect charge transfer kinetics within surface-bound porphyrins [14], etc.

In this context, one of particularly interesting features of tetrapyrrolic chemistry is the coordination-induced spin crossover (CISCO) [15]. This phenomenon, as the name implies, involves switching of the spin states of the metal center upon increase of the coordination number (*CN*). On the one hand, the interest in this process is explained by its relevance to naturally occurring switching between S = 2 and 0 spin states upon binding of oxygen to iron cation in heme molecules [2]. On the other hand, compounds, which demonstrate such behavior, are becoming popular components of sensors, materials with tunable optical and magnetic properties, and spintronic devices [16,17]. Due to the clearly recognizable photophysical properties of porphyrinoids, this switching can be read out via various spectroscopic techniques, which probe electronic structure of the tetrapyrrolic macrocycle. Such spectroscopic studies can be especially important when direct magnetochemical measurements of the spin states are not available.

**Figure 1:**
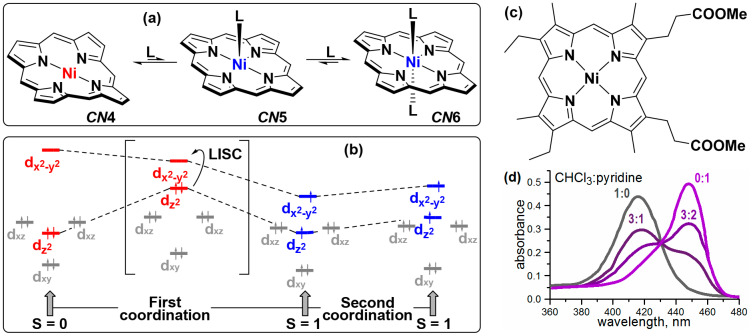
(**a**) axial coordination to Ni(II) porphyrinates, leading to formation of high-spin pyramidal and bipyramidal complexes with coordination numbers *CN* = 5 and 6; (**b**) partial molecular orbital scheme, depicting the electronic structure of low- and high-spin states of Ni(II) porphyrinates (adapted from Reference [23]); (**c**,**d**) Ni(II) complex with dimethyl ester of mesoporphyrin IX, for which axial coordination was observed for the first time, and corresponding spectral changes in the Soret band region upon increase of pyridine fraction in the solvent (adapted from Reference [19]).

Among tetrapyrrolic complexes, which exhibit CISCO behavior, special attention is paid to nickel(II) complexes. Nickel(II) porphyrinates lacking axial ligands are distorted macrocycles, where the metal center is square planar and diamagnetic (S = 0), but switches to paramagnetic state (S = 1) upon coordination of one or two axial ligands due to the difference in occupancies of $d_{x^{2}-y^{2}}$ and $d_{z^{2}}$ orbitals (Figure 1a,b). Importantly, binding of the first ligand flattens the macrocycle and also facilitates the binding of the second one. While archetypal Ni(II) complexes, such as octaethylporphyrinate, **NiOEP**, and tetraphenylporphyrinate, **NiTPP**, do not tend to coordinate additional ligands unless dissolved in almost pure N-donor solvents [18], specially tailored porphyrins can have much greater affinity for axial coordination, which obviously expands their possible application area.

Studies of axial coordination of N-donor ligands to nickel porphyrinates were started in early 1960′s on the example of Ni(II) complex with mesoporphyrin IX dimethyl ester, whose UV-vis spectra in chloroform and pyridine differed drastically, exhibiting ca. 30 nm bathochromic shift of the Soret band in coordinating solvent (Figure 1c,d); moreover, the latter solution showed clearly paramagnetic character [19]. Later, the influence of such coordination on conformational state of the porphyrin ring and various spectroscopic signatures of this influence were studied [18,20,21,22]. These studies emphasized the importance of electronic effects both in the porphyrin ring and in axial ligands to provide optimal balance between σ-donor versus π-acceptor bonding [15].

**Figure 2:**
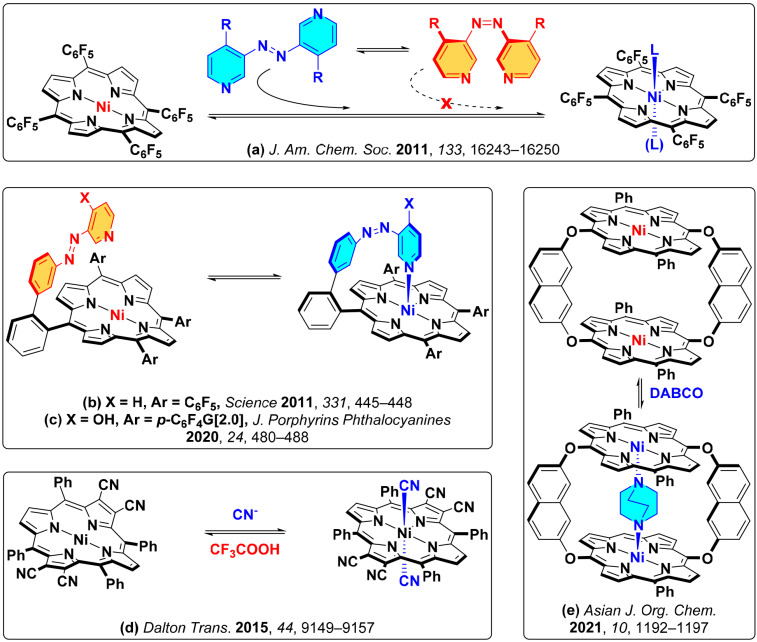
(**a**) coordination of photodissociable external ligands to Ni(II) tetra(pentafluorophenyl)porphyrinate [24]; (**b**) reversible light-induced magnetic switching of azopyridine functionalized Ni(II) porphyrin [25]; (**c**) pH-dependent spin switch, acting in aqueous solution; here G[2.0] stands for hydrophilic dendritic glycerol substituent [26]; (**d**) electron deficient Ni(II) porphyrinate, acting as a colorimetric “naked eye” selective sensor of CN^−^ anions [27]; (**e**) cofacial Ni(II) porphyrin dimer exhibiting efficient binding of 1,4-diazabicyclo[2.2.2]octane with Ni(II) metal centers [28].

A new round of research on Ni(II) multistable complexes began with the development of novel approach towards switching of magnetic properties of these molecules, namely light-induced spin change by photodissociable external ligands [24]. The authors used azopyridine ligands capable of photoisomerisation between *cis*- and *trans*-states, which in turn possessed different affinity to Ni(II) metal center in electron-deficient tetra(pentafluorophenyl)porphyrin complex (Figure 2a). The ratios of coordinated and noncoordinated forms at photostationary states could be regulated by bulkiness of substituents in the azopyridine ligands.

Following this principle, covalent attachment of azopyridine moieties to the porphyrin core afforded photoactive complexes exhibiting magnetic bistability in homogeneous solution at room temperature [25] (Figure 2b). Modification of the movable handle with electron-donating substituents allowed controlling the efficiency of light-induced spin state switching. For example, introduction of the ionizable OH-group afforded pH-responsive magnetic switch [26], whose compatibility with aqueous solutions was improved by introduction of G[2.0]-hydrophilic dendrimeric substituents (Figure 2c). In this context, further design of Ni(II) complexes with optimized electronic and sterical features allowed development of receptors capable of binding guest molecules [28] or important analytes such as, for example, cyanide anions [27] (Figure 2d,e).

These examples provide an important insight: if a potential axial ligand is forced onto the metal center by an external stimulus, binding becomes highly probable. At this point, a question arises, can one forcibly manipulate a porphyrin disc in such a way that nickel metal center would be in close proximity to a potential extra-ligand and bind with it without chemical modification and utilization of excessive concentrations?

**Figure 3:**
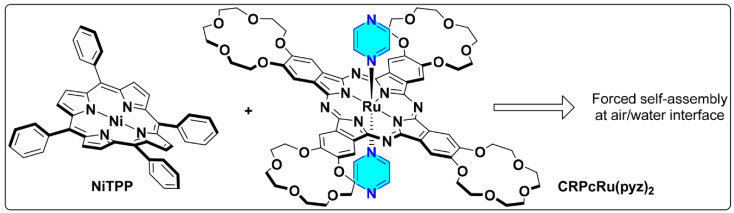
Components of a supramolecular garland, formed by forced coordination of pyrazine ligands to Ni(II) centers in the present work.

In the present work we study the possibility to achieve *CN*5 and *CN*6 states for a readily available nickel(II) tetraphenylporphyrinate, **NiTPP**, via forced axial coordination by pyrazine ligands incorporated into ruthenium(II) tetra-15-crown-5-phthalocyaninate, **CRPcRu(pyz)_2_** (Figure 3). We propose to achieve the necessary mutual orientation of the components by placing them at the air/water interface, and lateral compression will be used as the driving force for the controlled forced rapprochement of the nickel metal centers and the coordinating ligands.

## 2. Materials and Methods

Complexes **NiTPP** and **CRPcRu(pyz)_2_** were synthesized using the previously reported procedures [29,30]. Their chemical structures are shown in Figure 3. Right prior to the formation of the corresponding Langmuir monolayers, complexes were purified by column chromatography on neutral alumina.

The solutions of **NiTPP** and **CRPcRu(pyz)_2_** for spectral and Langmuir monolayer studies were prepared in distilled chloroform. Concentrations used were ca. 5 × 10^−6^ M for the nickel porphyrinate and ca. 1 × 10^−5^ M for the ruthenium complex. In case of mixed monolayers study, the component solutions were mixed in appropriate volumes to achieve a 1 to 1 molar ratio before the spreading onto the water surface.

Langmuir–Blodgett device KSV Minitrough (Espoo, Finland) with PTFE trough with surface area of 273.0 cm^2^ and moveable barriers made of hydrophilic polyacetal was used for Langmuir monolayer formation. Compression isotherms were recorded using automated Langmuir balance and platinum Wilhelmi plate. The monolayers were formed by spreading chloroform solutions onto the air/water interface using a chromatographic syringe. Then the system was left undisturbed for 10–15 min in order for the solvent to evaporate from the interface. After that, monolayer compression at the rate of 5 mm min^−1^ commenced. Expansion of compressed monolayers was carried out with the same rate. Ultrapure water (18 MΩ cm) deionized by a Milli-Q water purification system (Millipore, Burlington, MA, USA) was used as a subphase in Langmuir monolayer studies. All experiments were carried out at 25 °C in air.

In situ absorbance UV-vis spectra of monolayers on aqueous subphases were recorded in the reflection-absorption mode according to a previously described technique [31]. The measurements were carried out in the wavelength range of 350–900 nm using an AvaSpec-2048 fibre optic spectrometer equipped with a halogen light source AvaLight HAL (Avantes, Apeldoorn, The Netherlands). Light source wavelength range was limited by a 350 nm longpass filter to avoid harsh UV photodamage of the studied monolayers. The UV-vis reflectometric probe with a fibre diameter of 400 µm combined with a six-fibre irradiating cable was placed perpendicularly to the subphase surface at a distance of 2–3 mm from the monolayer. The signal obtained upon reflection of light from the subphase surface immediately before the monolayer spreading was used as a baseline.

## 3. Results

**Figure 4:**
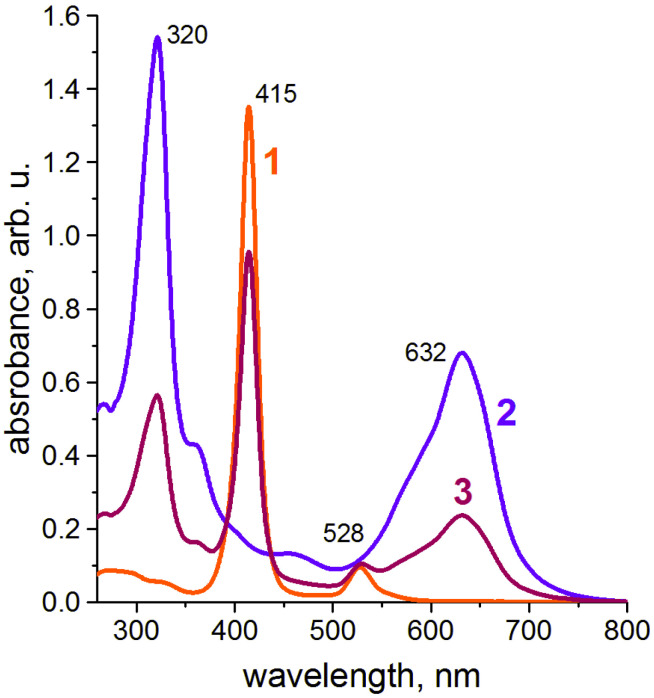
UV-vis spectra of (1) 5.1 × 10^−6^ M **NiTPP**, (2) 1 × 10^−5^ M **CRPcRu(pyz)_2_** solutions in chloroform, and an UV-vis spectrum of (3) the mixed solution of both in 1:1 molar ratio (concentration of each component is 3.4 × 10^−6^ M).

At the first stage of the present study, we have measured the UV-visible spectra of the **NiTPP** and **CRPcRu(pyz)_2_** chloroform solutions as precursors for Langmuir deposition. The obtained spectra are provided in Figure 4. Curve 1 shows characteristic UV-vis spectrum of a nickel tetraphenylporphyrinate in chloroform solution with a prominent Soret band ca 415 nm. Spectrum of **CRPcRu(pyz)_2_** (curve 2) in chloroform demonstrates a number of absorbance bands, characteristic to ruthenium crown-phthalocyaninates [32,33,34,35]. It should be noted that none of them overlap strongly with the **NiTPP** Soret band. As expected, UV-vis spectrum of a mixed solution with 1 to 1 molar ratio (Figure 4, curve 3) represents a sum of the component spectra, signifying that no chemical interactions take place between the porphyrinate and phthalocyaninate in the solution.

**Figure 5:**
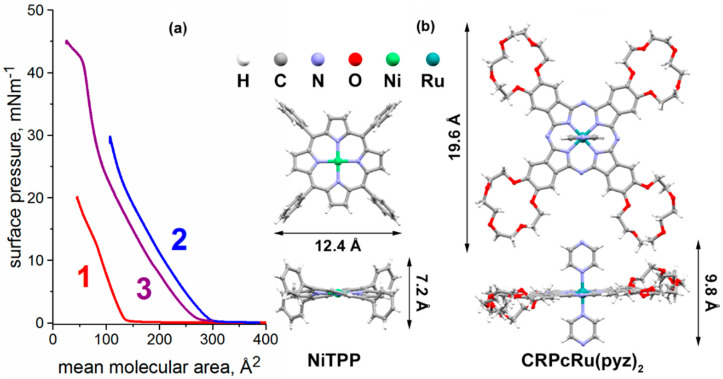
(**a**) Langmuir monolayer compression isotherms for (1) **NiTPP**, (2) **CRPcRu(pyz)_2_**, and (3) a mixed monolayer comprised of both in 1:1 molar ratio. (**b**) Chemical structures of the **NiTPP** and **CRPcRu(pyz)_2_** molecules obtained from X-ray crystallographic data [CCDC deposition numbers 904978 and 1056316 respectively] and corresponding estimated geometrical dimensions.

Continuing the study of the individual compounds in question, we have formed the monolayers of **NiTPP** and **CRPcRu(pyz)_2_** at the air/water interface. Obtained surface pressure–mean molecular area isotherms of these monolayers (Figure 5a, curves 1 and 2) both show typical forms for true Langmuir monolayers: regions, where compression does not lead to an increase of surface pressure at the right side of the graph, and a sharp bend at the limiting mean molecular area, where the molecules compressed in the monolayer start to interact with each other leading to subsequent growth of the surface pressure.

The isotherm for the **NiTPP** monolayer (Figure 5a, curve 1) exhibits a limiting molecular area value ca. 150 Å^2^. Curiously, square approximation of the **NiTPP** molecule with a side of 12.4 Å (Figure 5b) yields theoretical molecular area of ~154 Å^2^, which can indicate initial face-on orientation of the complex at the air/water interface. Moreover, the slight bend of the isotherm ca. 85 Å^2^ corresponds well to a theoretical area that can be occupied by **NiTPP** molecule on its edge (rectangular approximation with sides of 12.4 and 7.2 Å, and thus area of ~89 Å^2^), indicating that at this compression stage, the molecules assume edge-on orientation in the monolayer.

In the case of **CRPcRu(pyz)_2_** (Figure 5a, curve 2), the observed limiting area value amounts to ca. 300 Å^2^, which corresponds to neither smallest nor largest possible areas that a crown-phthalocyaninate molecule can occupy at the interface. Considering the fact that **CRPcRu(pyz)_2_** bears two axial pyrazine ligands, which are quite hydrophilic, face-on orientation of the molecule is most likely. The observed discrepancy between the experimental molecular area and the theoretical one is commonly explained by hydrophilic and labile nature of the crown-ether moieties, which can be partially immersed into the aqueous subphase and/or deformed slightly in the monolayer [36,37,38,39].

**Figure 6:**
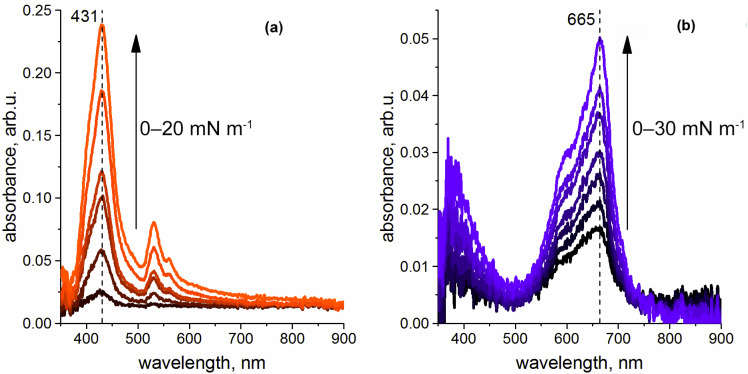
Evolution of the UV-vis spectra of the (**a**) **NiTPP** and (**b**) **CRPcRu(pyz)_2_** individual monolayers. Arrow on both panels denotes the direction of the spectral changes upon compression with the values of surface pressure denoted. Note a vertical dashed lane on panel (**a**) showing that the **NiTPP** Soret band ca. 431 nm does not shift during lateral compression in the individual monolayer.

Apart from certain bathochromic shifts of the whole spectra that arise due to change in polarity upon transfer from organic solvent phase to the air/water interface, the UV-vis spectra recorded in situ for the **NiTPP** and **CRPcRu(pyz)_2_** individual monolayers upon their compression (Figure 6a,b, respectively) exhibit only a gradual increase of the absorbance values with decrease of the mean molecular area values. This is explained by the increase of the density of the light absorbing molecules per unit of area at the interface upon lateral compression of the monolayer. No shifts of the initial absorbance bands nor appearance of new bands are observed upon compression. This means that no significant changes in aggregation state of the studied complexes in their individual monolayers, let alone the coveted change of the nickel metal center coordination number, take place in these monolayers, as was well expected.

Having built the foundation in form of the data on individual monolayers, we can move on to the exciting part of this study, i.e., the mixed Langmuir monolayers of **CRPcRu(pyz)_2_** and **NiTPP**. As was mentioned before, the UV-vis spectrum of a solution of these two complexes in chloroform (from which the Langmuir monolayers are eventually formed) shows no shifts of the absorbance bands, indicating absence of any interaction of the components in the solution (Figure 4).

Deposition of this solution onto the air/water interface as per Langmuir technique and subsequent compression of the resulting monolayer leads to some interesting observations. First of all, compression isotherm of the **NiTPP** and **CRPcRu(pyz)_2_** mixed monolayer (Figure 5a, curve 3) differs drastically from the theoretical one for non-interacting components. As it is a mixed monolayer, the mean molecular area values represent area occupied by some ‘average’ molecule in the monolayer. The experimental limiting mean molecular area value for the this mixed monolayer amounts to ca. 280 Å^2^.

In order to analyze this value somehow, it is convenient to compare it to the molecular areas of the individual components, as observed in the respective monolayer compression isotherms. To do so, we can employ the following formulas:

(1) $$A_{mix}^{th}=x\cdot A_{NiTPP}^{ind}+\left(1-x\right)\cdot A_{CRPcRu{\left(Pyz\right)}_{2}}^{ind}$$

(2) $$\Delta A_{mix}=A_{mix}^{th}-A_{mix}^{th}$$

where $A_{mix}^{th}$ is the calculated theoretical limiting molecular area occupied by imaginary average molecule of the mixed monolayer assuming no interaction between the individual components occurs in the monolayer; $A_{NiTPP}^{ind}$ and $A_{CRPcRu{\left(Pyz\right)}_{2}}^{ind}$ are limiting molecular areas of **NiTPP** and **CRPcRu(pyz)_2_** observed in respective individual monolayers; *x* is the molar fraction of the first component, in our case with 1:1 molar ratio, this fraction amounts to 0.5; $A_{mix}^{exp}$ is the experimental limiting area observed for the mixed monolayer; and $\Delta A_{mix}$ is the difference between the experimentally observed and theoretically ideal areas for the mixed monolayer.

Plugging the corresponding values into Formulas (1) and (2) gives $\Delta A_{mix}$ of +55 Å^2^. Positive sign of this value indicates that repulsive interaction between the components of the mixed monolayer takes place. Quite significant positive value also indicates that no initial interaction between **NiTPP** and **CRPcRu(pyz)_2_** occurs before further compression. The logic being that, if the complexes were to form a coordination bond, they would have needed to assume edge-on orientation and approach each other; and thus, a negative $\Delta A_{mix}$ would have been observed, which is not the case here.

**Figure 7:**
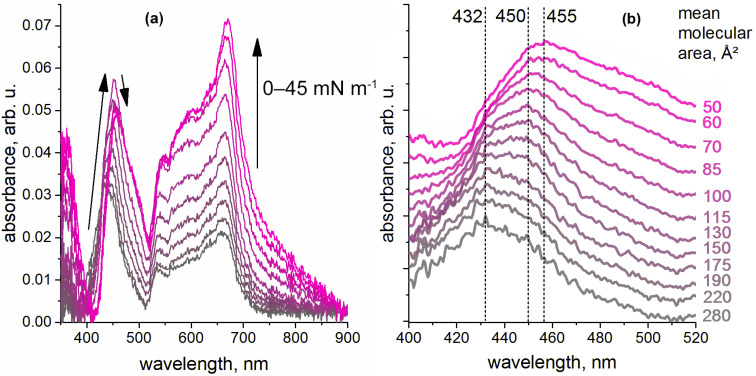
(**a**) Evolution of the UV-vis spectra of the **NiTPP ×** **CRPcRu(pyz)_2_** mixed monolayer upon its lateral compression; (**b**) Detailed view of the **NiTPP** Soret band: the spectra were normalized [0;1] and are presented here with a *y*-axis shift of 0.1 after each subsequent spectrum for clarity. Numbers to the right provide the mean molecular area values corresponding to the spectra.

Lateral compression of **NiTPP ×** **CRPcRu(pyz)_2_** mixed monolayer leads to a peculiar evolution of its UV-vis spectra (Figure 7a). Initially, absorbance bands of both components are in the positions, where they have been observed for the individual systems: **NiTPP** Soret band maximum is located ca. 432 nm, and Q-bands of both compounds are superimposed in the range from ca. 530 to 700 nm, with a dominating **CRPcRu(pyz)_2_** band at 663 nm. While for **CRPcRu(pyz)_2_** bands only a progressive increase of the absorbance intensity is observed upon compression of this mixed monolayer (as was the case for individual monolayers as well), indicating absence of any changes happening to this complex, evolution of the **NiTPP** Soret band is much more interesting. Figure 7b shows it in a clearer fashion.

It can be seen that initially the dominant wavelength of this band is 432 nm, and a small shoulder at 450 nm is already observed at the compression to the limiting mean molecular area of the mixed monolayer (ca. 280 Å^2^). At this mean molecule area, the molecules are already in contact in the monolayer, and we can assume that some of them are involved in coordination interactions. Further compression of the **NiTPP ×** **CRPcRu(pyz)_2_** mixed monolayer leads to redistribution of these bands in favor of the latter, which becomes a dominant sharp band at the monolayer compression stage with mean molecular area value ca. 115 Å^2^ (or with surface pressure of 19–20 mNm^−1^). Quite intriguing, this shift of the Soret band does not stop at this point. Further compression of the mixed monolayer leads to a broadening and long-wave shift of this band, which ends in the formation of a new absorption band with a maximum at 455 nm with a simultaneous small loss of absorbance value.

**Figure 8:**
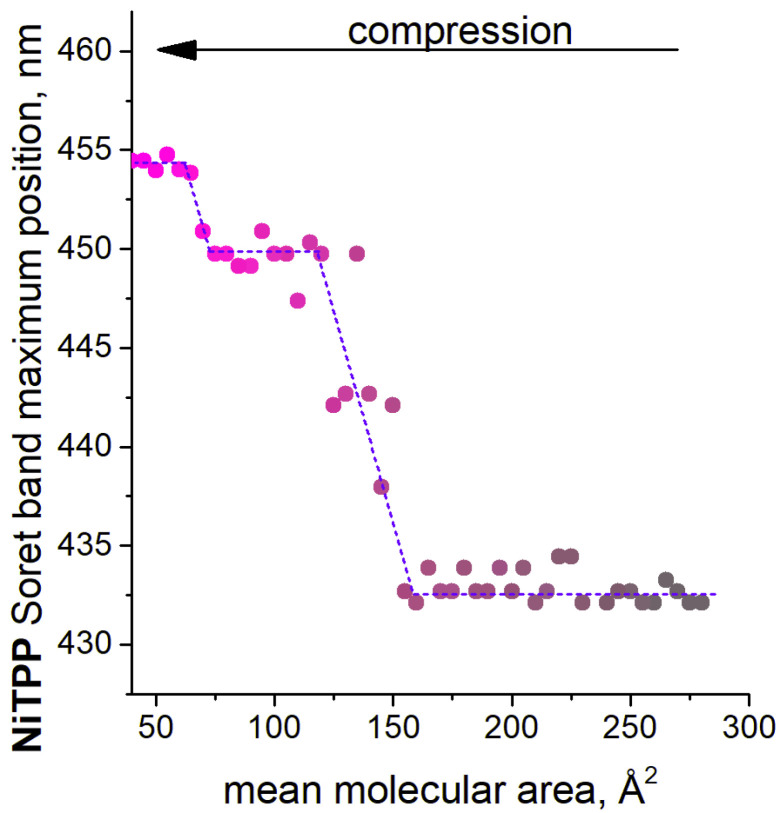
Dependence of the observed Soret band dominant wavelength on mean molecular area value of the mixed **NiTPP ×** **CRPcRu(pyz)_2_** Langmuir monolayer upon its lateral compression. The arrow denotes the direction of the compression (rapprochement of the molecules in the monolayer) for clarity.

These observations can be conveniently summarized as a dependence of the Soret band maximum absorbance wavelength on mean molecular area value (and thus, corresponding surface pressure). Such a graph is provided in Figure 8. It can be seen that the evolution of this band during the mixed monolayer compression is a stepwise process: at the first stage, the Soret band does not change and stays at ca. 432 nm, then a bathochromic shift by 18 nm occurs and is further followed by another 5 nm shift. These spectral changes are indubitably explained by axial coordination of the nickel center in the porphyrinate, first, in one axial position, and then in the second. As almost exactly the same bathochromic shifts were observed upon formation of the *CN*5 and *CN*6 complexes in the literature [40,41,42]. Notably, the shifts reported in the literature are almost identical numerically, and first extra-coordination leads to a large shift, while the second is accompanied by only several nanometers shift; exactly like in the data presented here. Clearly, the spectral shifts observed in our case are indicative of nickel metal center consistently attaining *CN*5 and *CN*6 upon compression in **NiTPP ×** **CRPcRu(pyz)_2_** mixed monolayer.

Another interesting observation is that in the mixed monolayer, usually strong Soret band of **NiTPP** does not dominate the spectra at later stages of compression. This, together with loss of absorbance of the band upon its shift mentioned above, can be considered another evidence for occurring interaction between **NiTPP** and **CRPcRu(pyz)_2_**, which is probably explained by differences in molar extinction of nickel complexes with *CN*4, *CN*5, and *CN*6.

**Figure 9:**
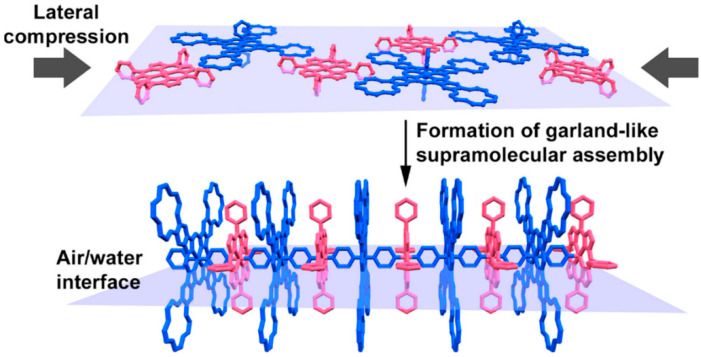
A cartoon illustrating the proposed formation of a supramolecular assembly of **NiTPP** and **CRPcRu(pyz)_2_** in the mixed Langmuir monolayer upon its lateral compression.

Thus, the observed behavior is highly likely due to formation of biaxial coordination compound between **NiTPP** and **CRPcRu(pyz)_2_** in the compressed monolayer. At the later stages, when the Soret band shift is maximal, indicating that *CN*6 nickel metal center is present in the system, most of the monolayer is probably comprised of extensive coordination polymers, in which each nickel metal center is bound with two ruthenium metal centers via pyrazine linkers. This is achieved only at such molecular area values, where both porphyrinate and phthalocyaninate molecules assume edge-on positions, and, due to close packing of the monolayer, make the closest possible contact between the coordinating pyrazine moiety and the nickel atom, thus, forcing the axial ligation. The general concept is illustrated by the cartoon in Figure 9 to clearly convey the nature of the hypothesized supramolecular assemblies. It is notable, that in contrast to previously reported cases, *CN*6 nickel in the present work was observed in a chemically unmodified tetraphenylporphyrinate complex.

Unfortunately, direct confirmation of the nickel metal center spin state, for example via EPR technique, is not possible at the air/water interface, and all our attempts to transfer the studied mixed monolayers onto solid substrates via Langmuir-Blodgett or Langmuir-Schaefer deposition have shown that in the film on solid substrate metal center in the porphyrinate complex returns to a mundane *CN*4 state. This is probably due to the extremely metastable nature of the system in question, as it exists in the unusual *CN*5 or *CN*6 state in form of Ni···Ru dimers or Ni···Ru···Ni···Ru garland-like coordination polymers in a compressed Langmuir monolayer, and any disturbances, like movement of a substrate through it, lead to local relaxations of the supramolecular structure and release of the **NiTPP** back into conformation with *CN*4 nickel center.

**Figure 10:**
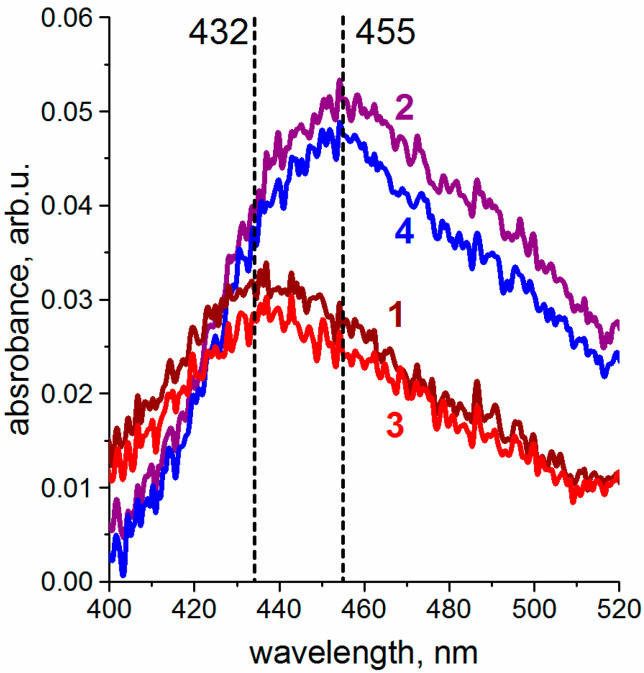
**NiTPP** Soret band upon lateral compression and expansion of **NiTPP ×** **CRPcRu(pyz)_2_** mixed monolayer. Presented spectra correspond to the first cycle of lateral compression from mean molecular area of (1) 220 Å^2^ to (2) 60 Å^2^, (3) monolayer expansion to 220 Å^2^, and (4) another compression to 60 Å^2^.

On the other hand, this exact dynamic behavior allows for control of nickel spin state in the mixed **NiTPP ×** **CRPcRu(pyz)_2_** Langmuir monolayer, since expansion of the compressed system also leads to reversal of the forced coordination, as evidenced by return of the **NiTPP** Soret band back to its initial position upon decompression (Figure 10). Notably, these spectral changes are repeatable under compression-expansion cycling.

This proof-of-concept study shows the possibility to tune the magnetic properties of nickel porphyrinates using a methodology unexplored before. Moreover, because the present phenomenon takes place at air/water interface, this brings potential application of such CISCO-enabled systems closer, as soft matter can be considered much more practical than bulk solutions in organic solvents. Since porphyrins and phthalocyanines, being redox-active ligands, exhibit extremely diverse and rich electrochemical properties, further development of this approach to control of metal center spin state can potentially lead to creation of systems, where redox-activity can be fine-tuned via CISCO and vice versa, in the future.

## 4. Conclusions

In the present work we show that *CN*5 and *CN*6 nickel(II) in an unmodified tetraphenylporphyrin can be achieved via forced axial coordination with a dipyrazine ruthenium(II) crown-phthalocyaninate, where the latter is used as a molecular scaffold that aligns nickel cations within the pyrazine-ruthenium-pyrazine coordination axis at the air/water interface. Lateral compression of these molecules at the water surface leads to their rapprochement and initiation of the nickel-pyrazine binding, which in its turn results in formation of bimolecular dimers with pentacoordinate Ni(II) center at the first stage, and then to further appearance of garland-like supramolecular heterometallic polymer, where nickel metal center assumes coordination number six. These transformations were found to be reversible, and nickel metal center can be prompted to release the extra-ligands upon relaxation of the mixed monolayer.

## Data Availability

The data presented in this study are available on request from the corresponding authors. The data are not publicly available due to the conditions of the funding body.

## Sample Availability

Samples of the compounds are available from the authors on request.
